# Machine learning model for predicting ciprofloxacin resistance and presence of ESBL in patients with UTI in the ED

**DOI:** 10.1038/s41598-023-30290-y

**Published:** 2023-02-25

**Authors:** Hyun-Gyu Lee, Youngho Seo, Ji Hye Kim, Seung Baik Han, Jae Hyoung Im, Chai Young Jung, Areum Durey

**Affiliations:** 1 Department of Medical Education and Medical Humanities, College of Medicine, Inha University, Incheon, Republic of Korea; 2 Department of Emergency Medicine, College of Medicine, Inha University Hospital, Inha University, 27, Inhang-ro, Jung-Gu, Incheon, 22332 Republic of Korea; 3 Department of Internal Medicine, College of Medicine, Inha University, Incheon, Republic of Korea; 4 Biomedical Research Institute, Inha University Hospital, Incheon, Republic of Korea

**Keywords:** Infectious diseases, Antimicrobials, Bacteria

## Abstract

Increasing antimicrobial resistance in uropathogens is a clinical challenge to emergency physicians as antibiotics should be selected before an infecting pathogen or its antibiotic resistance profile is confirmed. We created a predictive model for antibiotic resistance of uropathogens, using machine learning (ML) algorithms. This single-center retrospective study evaluated patients diagnosed with urinary tract infection (UTI) in the emergency department (ED) between January 2020 and June 2021. Thirty-nine variables were used to train the model to predict resistance to ciprofloxacin and the presence of urinary pathogens’ extended-spectrum beta-lactamases. The model was built with Gradient-Boosted Decision Tree (GBDT) with performance evaluation. Also, we visualized feature importance using SHapely Additive exPlanations. After two-step customization of threshold adjustment and feature selection, the final model was compared with that of the original prescribers in the emergency department (ED) according to the ineffectiveness of the antibiotic selected. The probability of using ineffective antibiotics in the ED was significantly lowered by 20% in our GBDT model through customization of the decision threshold. Moreover, we could narrow the number of predictors down to twenty and five variables with high importance while maintaining similar model performance. An ML model is potentially useful for predicting antibiotic resistance improving the effectiveness of empirical antimicrobial treatment in patients with UTI in the ED. The model could be a point-of-care decision support tool to guide clinicians toward individualized antibiotic prescriptions.

## Introduction

Urinary tract infection (UTI) is an extremely common condition encountered in the emergency department (ED). *Escherichia coli* accounts for 75–95% of bacterial isolates in community-onset UTI, followed by *Klebsiella pneumonia* and *Proteus mirabilis*^1^. The increasing resistance of these uropathogens to commonly used antimicrobials for UTI has been a concerning global issue. For instance, the resistance rate of *E. coli* to fluoroquinolones, especially ciprofloxacin, ranges from 55.5% to 85.5% in developing countries and from 5.1 to 32% in developed countries^2^. The incidence of extended-spectrum beta-lactamases (ESBL) -producing *E. coli* and *K. pneumonia* infections has also increased in the United States, limiting treatment options of antibiotics as ESBL is capable of degrading most penicillins, cephalosporins, and aztreonams^3,4^.

Increasing resistance of uropathogens is a clinical challenge to emergency physicians because antibiotics in the ED are selected before an infecting pathogen or its antibiotic resistance profile is confirmed. The challenge in this empiric therapy is the use of effective antibiotics for which the pathogen is susceptible to. Given that ineffective initial antimicrobial therapy leads to prolonged hospitalisation and increased medical costs and mortality^5,6^, starting effective antibiotics in the ED which, the pathogen is susceptible is of vital importance.

Electronic decision support systems in conjunction with machine learning (ML) algorithms could reduce this burden of emergency physicians during empiric treatment and have thus been gaining attention. Despite the high quality of many of these studies, there were overlapping limitations such as a lack of patients’ clinical variables like comorbidities, vital signs or laboratory data, generalization of heterogeneous data from multiple infection sites or from multiple bacterial species, use of a methodology of logistic regression models, or unperformed importance analysis or feature selection^7–13^. Also, they have not yet been actively used in clinical practice owing to a lack of integration into clinical work^14^.

Thus, this study aimed to develop a practical prediction model for antibiotic resistance in UTI patients, using ML algorithms. Towards this goal, we analysed the decision-making process from the physician’s perspective and created a predictive model using Gradient-Boosted Decision Tree (GBDT) that could be easily integrated into clinical practice to, ultimately, improve the effectiveness of empiric antibiotic therapy in the ED.

## Materials and methods

### Ethics

This study was approved by the Institutional Review Board of Inha University Hospital, Incheon, Korea (IRB no. 2021-08-016-005) and was conducted according to the Declaration of Helsinki. The need for informed consent was waived by the Institutional Review Board of Inha University Hospital owing to the retrospective nature of the study.

### Study design and population

This was a single-center retrospective study with patients diagnosed with UTI in the ED between January 2020 and June 2021. This study was conducted in a tertiary university hospital in Korea with an average of 60,000 patients according to an annual census of ED visits. The electronic medical records (EMRs) of all adult patients (age ≥ 18 years) who were diagnosed with UTI and had positive urine culture (≥ 10^5^ CFU/mL) over the 18 months were reviewed. Patients with UTI with multiple pathogens were excluded because the growth of two or more urinary pathogens is generally considered as contamination^15^, and only data from the index admission were included in the analysis for patients with multiple admissions during the study period.

### Data collection and definition

Total of 39 variables were used to train the predictive model with a focus on resistance to ciprofloxacin and the presence of ESBL of urinary pathogens (Table 1). Majority of them have been suggested in previous studies to be related to either resistance to ciprofloxacin or the presence of ESBL of urinary pathogens, and some variables which could influence on the resistance pattern were included reflecting authors’ opinions.

**Table 1:** Thirty-nine variables used to train the predictive model.

Variables	
Age	Vital signs on presentation
Gender	Systolic blood pressure
Bed-ridden	Diastolic blood pressure
Mental change	Pulse rate
Nursing home residence	Respiratory rate
	Body temperature
Medical device	Peripheral oxygen saturation
Urinary catheter/Cystostomy	
	Complete blood cell counts
Infection type	Leukocyte count
Hospital-acquired	Hemoglobin
	Platelet
Comorbid conditions	
Diabetes mellitus	
Hypertension	Other laboratory findings
Cardiovascular disease	C reactive protein
Chronic renal failure	Glucose
Cerebrovascular disease	Blood urea nitrogen
Malignancy	Creatinine
Past history	Arterial blood gas analysis
Hospitalization within 3 months	pH
Antibiotics use within 3 months,	PCO_2_
UTI within a year	PO_2_
	HCO_3_^−^
Assessment models	Arterial oxygen saturation
qSOFA	Lactic acid
Hypothermia	
Leukopenia	
Medication	
Antacid, H_2_ blocker or proton pump inhibitor	

The dataset included the bacterial species isolated from the patients’ urine and their antimicrobial susceptibility (microbiology data), and clinical data (e.g., age, sex, comorbidities, vital signs, laboratory data, etc.). Infections were considered to be hospital-acquired if the patients were transferred to the ED from another hospital after 48-h hospitalisation or if the patient was discharged from the hospital within 3 days of the UTI diagnosis. We defined an episode of previous UTI within the year before the index date and used a 30-day time window to distinguish the index UTI episode from previous episodes. Hence, we considered it a relapse with the same uropathogen due to incomplete treatment rather than rapid re-infection and excluded it when the patient presented with UTI within the 30-day time window^16^.

All prescriptions within 3 months before the index UTI were identified by reviewing medical records from prior/current hospitalisations and hospital visits including medication from other hospitals for current use of gastric mucosa-protecting agents (histamine-2 receptor blocker, proton pump inhibitor, and antacids) and for history of antibiotic use. The quick Sequential Organ Failure Assessment score was calculated using the following criteria: systolic blood pressure ≤ 100 mmHg, respiratory rate ≥ 22/min, and altered mental status^17^. Each criterion corresponded to 1 point, with the total score ranging from 0 to 3. Hypothermia was defined as body temperature < 36 °C and leukopenia as a white blood cell count < 4000/mm^3^ upon ED arrival. The result ‘intermediate’ on the antimicrobial susceptibility test was considered as ‘resistant’ in contrast with ‘susceptible’.

#### Bacterial identification and antimicrobial susceptibility

Fresh urine samples were placed on the eosin methylene blue agar, and the isolated microorganisms were categorized by Gram staining. Bacteria were identified using the VITEK2 system (bioMérieux, Marcy l'Etoile, France). Antibiotic susceptibilities were tested using the disk diffusion method in accordance with the criteria from the Clinical and Laboratory Standards Institute.

#### Testing for ESBL phenotype

ESBL confirmatory test involved testing cefotaxime (30 μg) and ceftazidime (30 μg) alone and in combination with clavulanate (10 μg) on Mueller–Hinton agar. If the zone diameter increased ≥ 5 mm in the presence of clavulanate, the isolate was considered ESBL-producing. *Escherichia coli* ATCC 25,922 and *Klebsiella pneumonia* ATCC700603 (700,603.18) were used as quality controls.

#### Effectiveness of antimicrobial agents

The effectiveness of initial empirical antimicrobial therapy with ciprofloxacin or third- or fourth-generation cephalosporins (cefotaxime and cefepime) in the ED was evaluated based on the results of in vitro antimicrobial susceptibility testing. For example, empirical treatments with third- or fourth-generation cephalosporins were considered as ‘ineffective’ when pathogens from urine culture were positive for ESBL phenotype (ESBL-producing pathogens).

### Interim result

A total of 550 UTI patients with an average age of 72.1 years were enrolled. Majority of the patients were female (n = 409, 74%). There were 208 (37%) patients who were bedridden and 71 (12%) patients who had an indwelling urinary catheter upon ED arrival. Underlying diabetes and neurologic diseases including stroke were identified in 231 (42%) and 238 (43%) patients, respectively. A history of previous UTI within one year was found in 130 (23%) patients, and 105 (19%) patients had hospital-acquired infections. For laboratory results, the average levels of C-reactive protein and creatinine were 12.12 mg/dL and 1.34 mg/dL, respectively.

**Figure 1:**
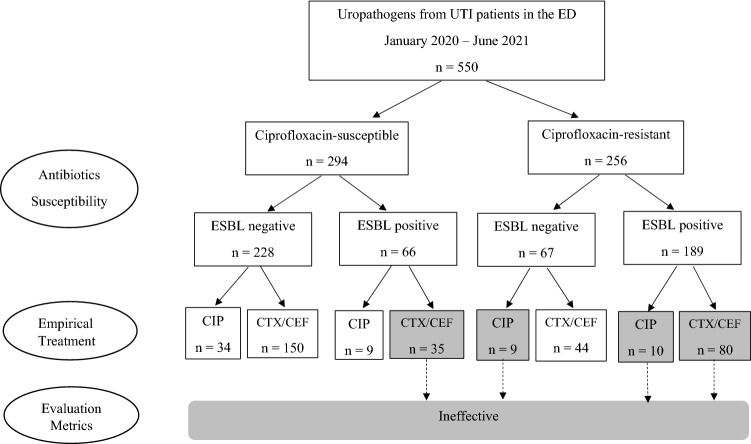
Antibiotics susceptibility of the uropathogens and empirical antibiotics used in the emergency department (ED).

The most common bacteria grown in urine cultures was *E. coli* (91%), followed by *Klebsiella* (8%). Overall, 46% of uropathogens showed resistance to ciprofloxacin, and the ESBL positivity rate was 46%, and IE rate in ET was 24% (134/550) (Fig. 1). The most common empirical antibiotics prescribed by emergency physicians was cefotaxime (55%), followed by piperacillin-tazobactam (28%) and ciprofloxacin (11%). The data distribution with mean values of variables and data missing rates was summarized in Table 2 showing well balance data and insignificant missing rates.

**Table 2:** Selected variables during customization each for predicting ciprofloxacin (CIP) resistance and ESBL positivity with data distribution.

Selected variables	Training sets (2020)				Testing sets (2021)			
	CIP-susceptible (n = 228)		CIP-resistant (n = 195)		CIP-susceptible (n = 66)		CIP-resistant (n = 61)	
	Mean (SD)	Missing rate (%)	Mean (SD)	Missing rate (%)	Mean (SD)	Missing rate (%)	Mean (SD)	Missing rate (%)
Hospital-acquired (%)	7.5	0	31.8	0	3.0	0	39.3	0
Bedridden (%)	23.2	0	53.8	0	16.7	0	63.9	0
1y-UTI	14.5	1.8	37.9	0.4	6.1	0.4	31.1	0.4
DBP	74.8(18.1)	0	70.3(17.1)	0	74.4(17.9)	0	73.3(18.5)	0
Platelet	202.2(99.4)	0	222.7(100.2)	0	197.5(107.9)	0	208.9(99.3)	0
BT	38.2(1.2)	0	37.7(1.2)	0	43.2(43.1)	0	37.4(1.1)	0
pCO_2_	32.9(7.3)	18.0	33.2(6.8)	6.6	34.5(6.9)	6.1	35.5(6.8)	1.3
Creatinine	1.3(1.6)	0	1.5(1.2)	0	1.2(0.8)	0	1.2(0.8)	0
SpO_2_	95.6(5.8)	4.8	95.0(4.1)	1.8	97.0(3.0)	1.3	96.3(2.6)	0.9
SaO_2_	95.0(4.1)	6.6	93.6(6.9)	1.8	110.2(113.1)	3.1	94.7(3.6)	1.3
3 m-admission (%)	24.1	1.3	51.3	0.4	13.6	0.4	60.7	0.4
Cerebrovascular (%)	31.1	0	54.9	0	40.9	0	54.1	0
HCO_3_	22.8(4.7)	18.0	23.6(7.8)	6.6	23.9(4.2)	6.1	25.6(10.8)	1.3
SBP	129.9(29.9)	0	123.8(30.0)	0	130.3(28.9)	0	128.0(35.2)	0
3 m-antibiotics (%)	15.4	1.8	32.8	0.4	7.6	0.4	37.7	0.4
WBC	11,751.0 (5875.1)	0	12,640.6 (7263.6)	0	11,760.9 (6988.1)	0	11,204.1 (5453.1)	0
Mental change (%)	18.9	0	23.1	0	7.6	0	34.4	0
PR	100.3(21.3)	0	96.3(22.0)	0	99.3(23.2)	0	92.7(22.9)	0
pH	47.4(546.6)	18.0	48.7(553.7)	6.6	7.4(0.1)	6.1	7.4(0.1)	1.3
Glucose	171.6(98.9)	0	160.9(85.1)	0	145.1(64.3)	0	175.4(125.5)	0

Selected variables	Training sets (2020)				Testing sets (2021)			
	ESBL negative (n = 225)		ESBL positive (n = 198)		ESBL negative (n = 70)		ESBL positive (n = 57)	
	Mean (SD)	Missing rate (%)	Mean (SD)	Missing rate (%)	Mean (SD)	Missing rate (%)	Mean (SD)	Missing rate (%)
3 m-admission (%)	19.6	0.4	56.1	1.5	15.7	2.9	61.4	0
3 m-antibiotic (%)	9.8	0.9	38.9	1.5	8.6	2.9	38.6	0
Bedridden (%)	22.7	0	54.0	0	18.6	0	64.9	0
Cerebrovascular (%)	29.3	0	56.6	0	35.7	0	61.4	0
BUN	28.8(64.6)	0	29.7(21.6)	0	25.3(20.3)	0	25.3(14.2)	0

SD, standard deviation; 1y-UTI, UTI within a year; DBP, diastolic blood pressure; BT, body temperature; SpO2, peripheral oxygen saturation; SaO2, arterial oxygen saturation; 3 m-admission, hospitalization within 3 months; SBP, systolic blood pressure; 3 m-antibiotics, antibiotics use within 3 months; WBC, leukocyte count; PR, pulse rate.

### Machine learning process

#### Data structure and model building

**Figure 2:**
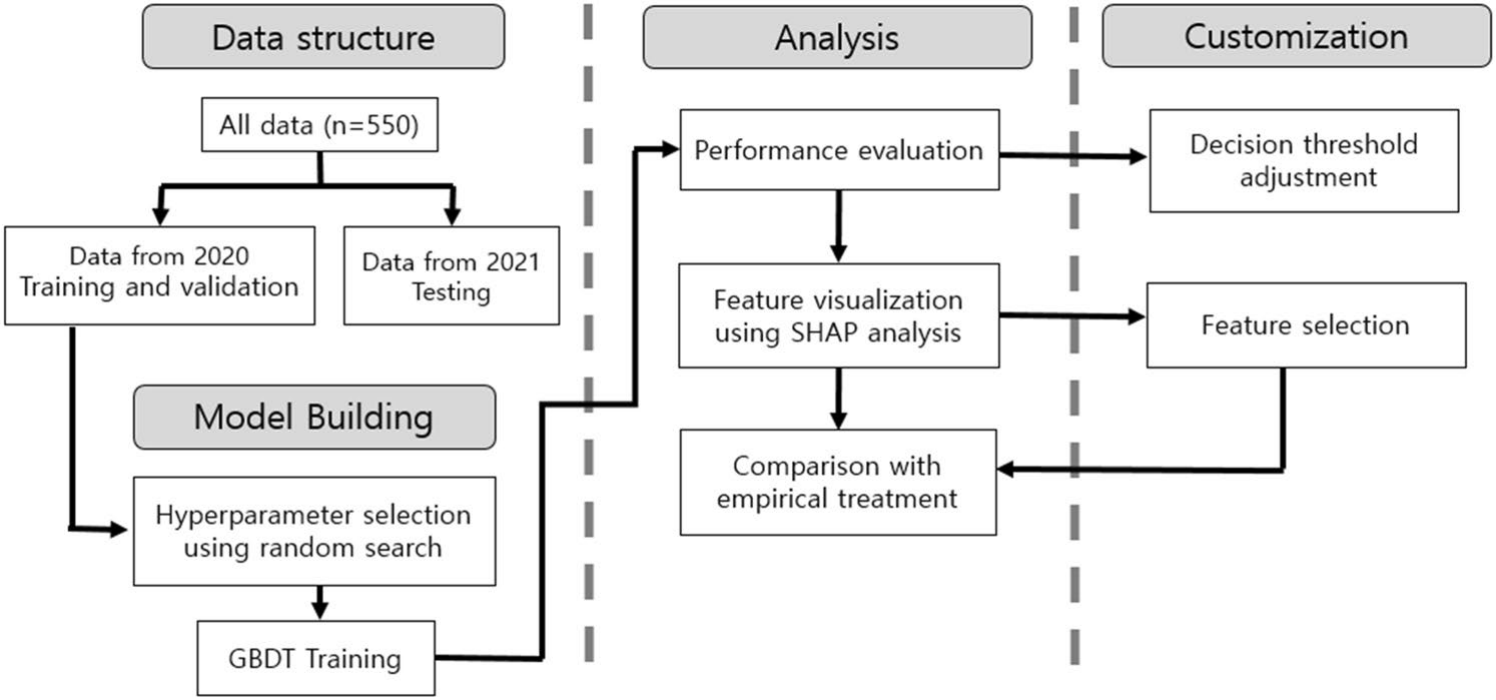
Abstract diagram of this study. GBDT: gradient-boosted decision tree, SHAP: Shapley additive explanations.

Machine learning process in our study was overviewed in Fig. 2. Considering that antibiotic resistance changes over time, data was split into 2020 and 2021, and data from the year of 2020 were used for training and validation while data from the year of 2021 was used for testing in order to reflect the most up-to-date trend of antibiotics resistance. Given that GBDT can both categorical and continuous variables without pre-processing, we used raw data to train the model without processes of imputation or scaling methods.

GBDT hyperparameter was selected using fivefold cross-validation with random search. We tried twenty random searches for maximum depth between 1 and 5, number of estimators between 100 and 500, and learning rate between 0.001 and 0.1. All experiments were performed on a PC with GeForce RTX 3090 Ti, Python 3.7.

#### Performance evaluation and visualization of feature importance

In the study, the model performance was evaluated using the area under the receiver operating characteristic curve (AUC), precision, sensitivity, and specificity.

$$\mathrm{Precision}=\frac{\mathrm{True\, positive \,samples}}{\mathrm{Samples\, predicted\, to\, be\, positive}}$$

$$\mathrm{Sensitivity}=\frac{\mathrm{Samples\, predicted\, to\, be\, positive}}{\mathrm{Actual\, positive \,samples}}$$

$$\mathrm{Specificity}=\frac{\mathrm{Samples\, predicted\, to \,be\, negative}}{\mathrm{Actual\,negative\, samples}}$$

Also, we visualized feature importance with 39 variables each for resistance to ciprofloxacin and presence of ESBL using SHapely Additive exPlanations (SHAP)^18^.

#### Comparison to empirical treatment

The GBDT model performance was compared with that of the original prescribers (emergency physicians) in the ED according to the effectiveness of the antibiotic selected. Because this study did not evaluate effectiveness of ET in patients who received antibiotics other than ciprofloxacin or third- or fourth-generation cephalosporins, model performance was validated by measuring the ineffectiveness, not the effectiveness. Ineffectiveness ($IE$) was calculated as follows:

$${IE}_{ET}=\frac{\left|\left\{ET=\mathrm{CIP}\right\}\cap \left\{{U}_{\mathrm{CIP}}=\mathrm{R}\right\}\right|+\left|\left\{ET=\mathrm{CTX}\right\}\cup \left\{ET=\mathrm{CEF}\right\}\cap \left\{{U}_{\mathrm{ESBL}}=\mathrm{P}\right\}\right|}{\left|\left\{{U}_{\mathrm{CIP}}=\mathrm{R}\right\}\cup \left\{{U}_{\mathrm{ESBL}}=\mathrm{P}\right\}\right|}$$

$${IE}_{GBDT}=\frac{\left|\left\{{GBDT}_{\mathrm{CIP}}=\mathrm{S}\right\}\cap \left\{{U}_{\mathrm{CIP}}=\mathrm{R}\right\}\right|+\left|\left\{{GBDT}_{\mathrm{ESBL}}=\mathrm{N}\right\}\cap \left\{{U}_{\mathrm{ESBL}}=\mathrm{P}\right\}\right|}{\left|\left\{{U}_{\mathrm{CIP}}=\mathrm{R}\right\}\cup \left\{{U}_{\mathrm{ESBL}}=\mathrm{P}\right\}\right|}$$

where CIP, CTX, and CEF indicate ciprofloxacin, cefotaxime, and cefepime, respectively.

$\left|\left\{ET=\mathrm{CIP}\right\}\cap \left\{{U}_{\mathrm{CIP}}=\mathrm{R}\right\}\right|$ was defined as the number of patients who were empirically treated with ciprofloxacin in the ED but the pathogens from urine cultures turned out to be resistant (R) to ciprofloxacin. Meanwhile, $\left|\left\{{GBDT}_{\mathrm{CIP}}=\mathrm{S}\right\}\cap \left\{{U}_{\mathrm{CIP}}=\mathrm{R}\right\}\right|$ was defined as the number of patients in whom the GBDT predicted their pathogens to be susceptible (S) to ciprofloxacin but were resistant on susceptibility testing. Given that this decision support tool by the GBDT would lead emergency physicians to select ciprofloxacin when ${GBDT}_{\mathrm{CIP}}=\mathrm{S}$, we treated $ET=\mathrm{CIP}$ and ${GBDT}_{\mathrm{CIP}}=\mathrm{S}$ equally when comparing the performance of the GBDT with ET. $\left\{{U}_{\mathrm{ESBL}}=\mathrm{N}\right\}$ and $\left\{{U}_{\mathrm{ESBL}}=\mathrm{P}\right\}$ indicated that the urine culture results were positive (P) or negative (N) for ESBL expression, respectively.

## Results

### GBDT model performance

**Figure 3:**
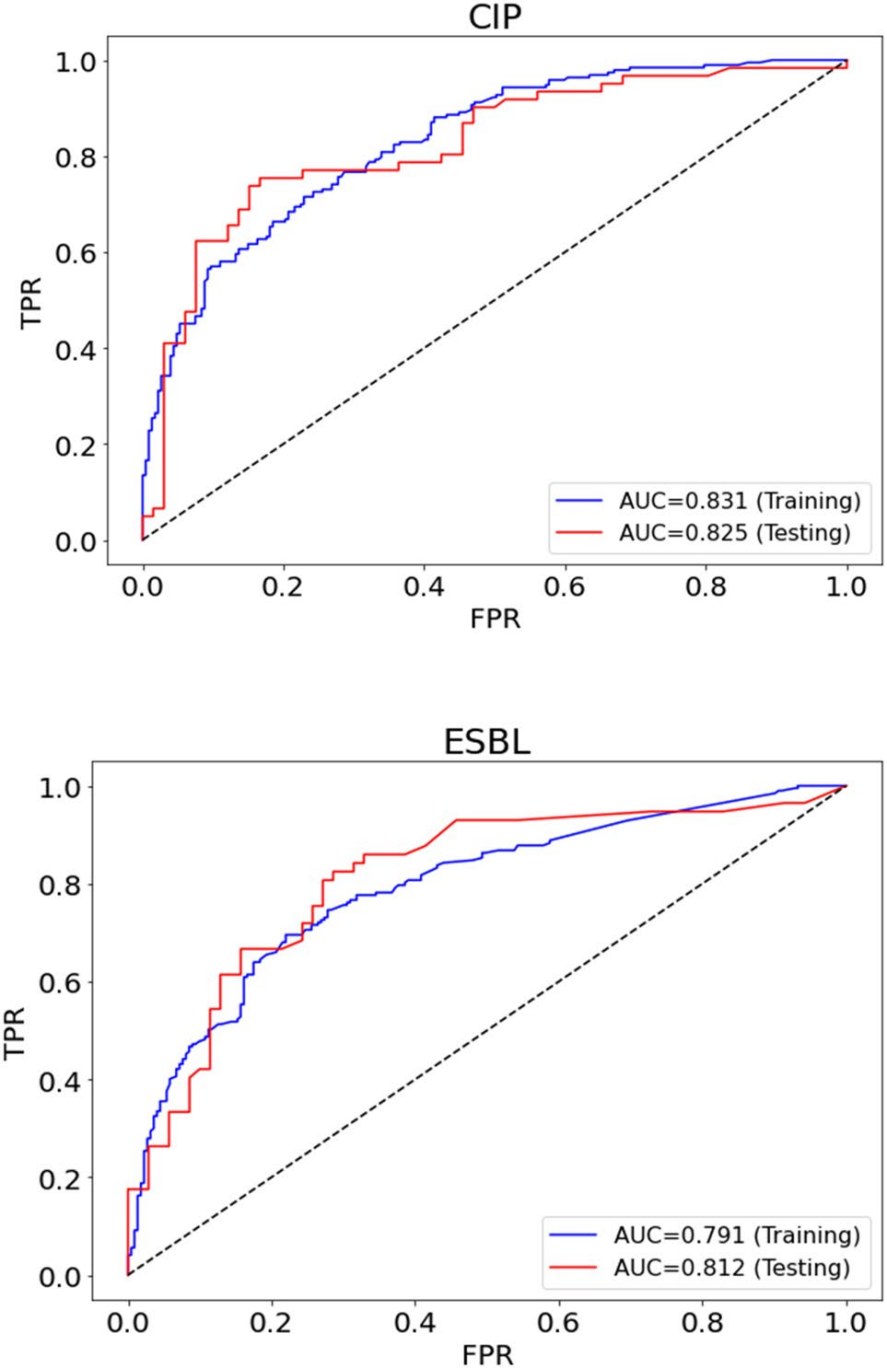
Overall performance of the Gradient-Boosted Decision Tree (GBDT) model. The area under the receiver operating characteristic curve (AUC) of training and testing sets in predicting resistance to ciprofloxacin (CIP) and the production of extended-spectrum β-lactamase* (*ESBL) of the uropathogen was shown. TPR is True Positive Rate, and FPR is False Positive Rate.

**Figure 4:**
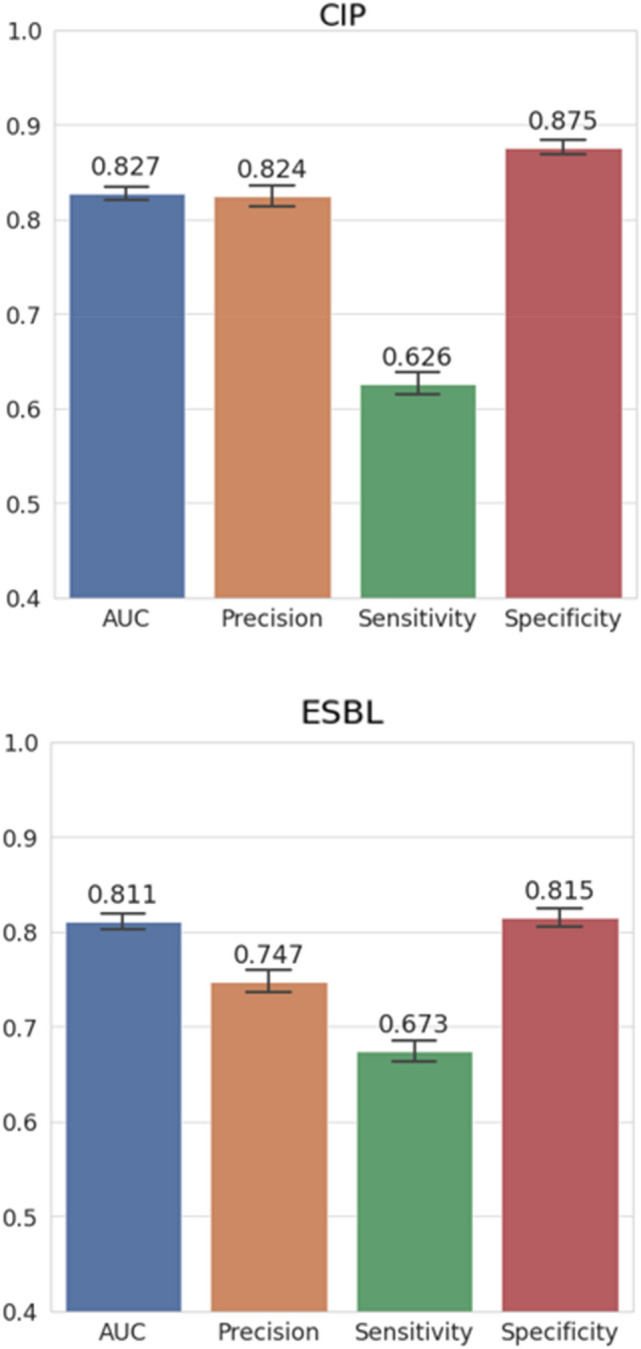
The classification performance of the Gradient-Boosted Decision Tree (GBDT). The decision threshold was set to 0.5 and 95% confidence intervals (error bars) were obtained by bootstrap analysis.

Overall performance of our GBDT model using AUC was shown in Fig. 3. No overfitting occurred in the model trained with 39 variables as AUCs for training and test sets were similar in predicting both CIP resistance and ESBL presence. The classification performance of GBDT using the AUC, precision, sensitivity, and specificity was summarized in Fig. 4. To estimate sampling error in the fixed test set, we measured 95% confidence intervals by bootstrap analysis using 100 times replacement sampling.

### Visualization of feature importance

**Figure 5:**
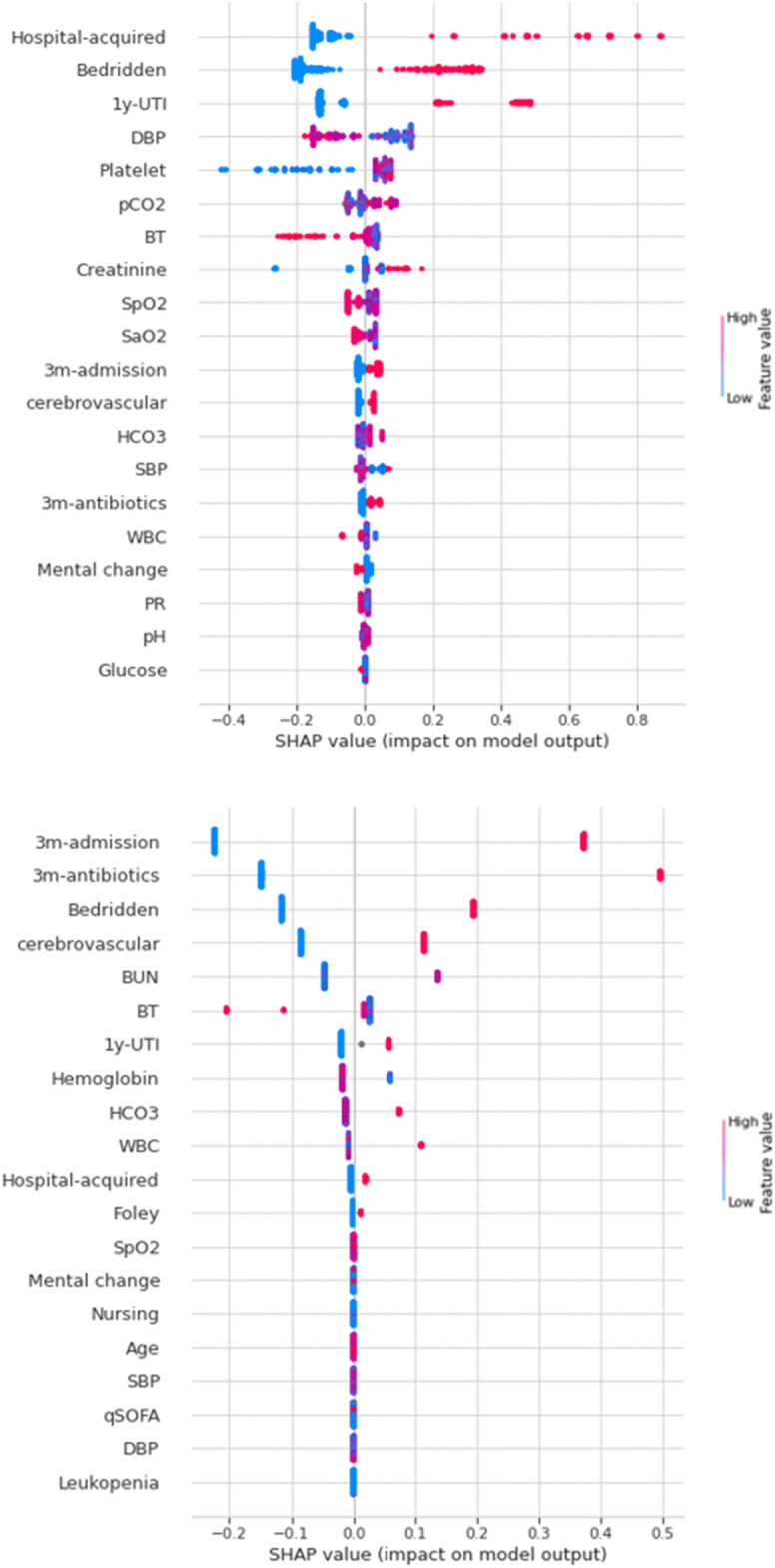
Feature importance for ciprofloxacin resistance (top) and positivity of extended-spectrum beta-lactamases (bottom) using Shapely Additive exPlanations (SHAP). The top 20 variables were visualized among 39 variables. 1y-UTI, UTI within a year; DBP, diastolic blood pressure; BT, body temperature; SpO2, peripheral oxygen saturation; SaO2, arterial oxygen saturation; 3m-admission, hospitalization within 3 months; SBP, systolic blood pressure; 3m-antibiotics, antibiotics use within 3 months; WBC, leukocyte count; PR, pulse rate.

Feature importance visualized using SHAP was shown in Fig. 5. Higher placed variables on the graph had higher SHAP feature values reflecting stronger influence on the model performance. The top-placed variables with higher importance in predicting resistance to CIP of uropathogens were presence of hospital-acquired infection, history of UTI within a year, bedridden status, lower initial diastolic blood pressure, and lower platelet counts. As for predicting positivity for ESBL phenotype, past history of hospitalization or antibiotic use within the last 3 months, bedridden status, and having cerebrovascular disease were shown to have higher SHAP feature values.

### Customizations of the model

Our final model was made through 2-step customization process. First, we customized the initial GBDT model through adjusting the decision threshold on the purpose of reducing IE. As shown in Fig. 4, the specificity of the initial model was higher than the sensitivity in predicting both CIP resistance and ESBL presence. Considering the importance of reducing the use of ineffective antimicrobials in the ED, we set the decision threshold for target to a lower value than 0.5, which is used generally in prediction models.

**Figure 6:**
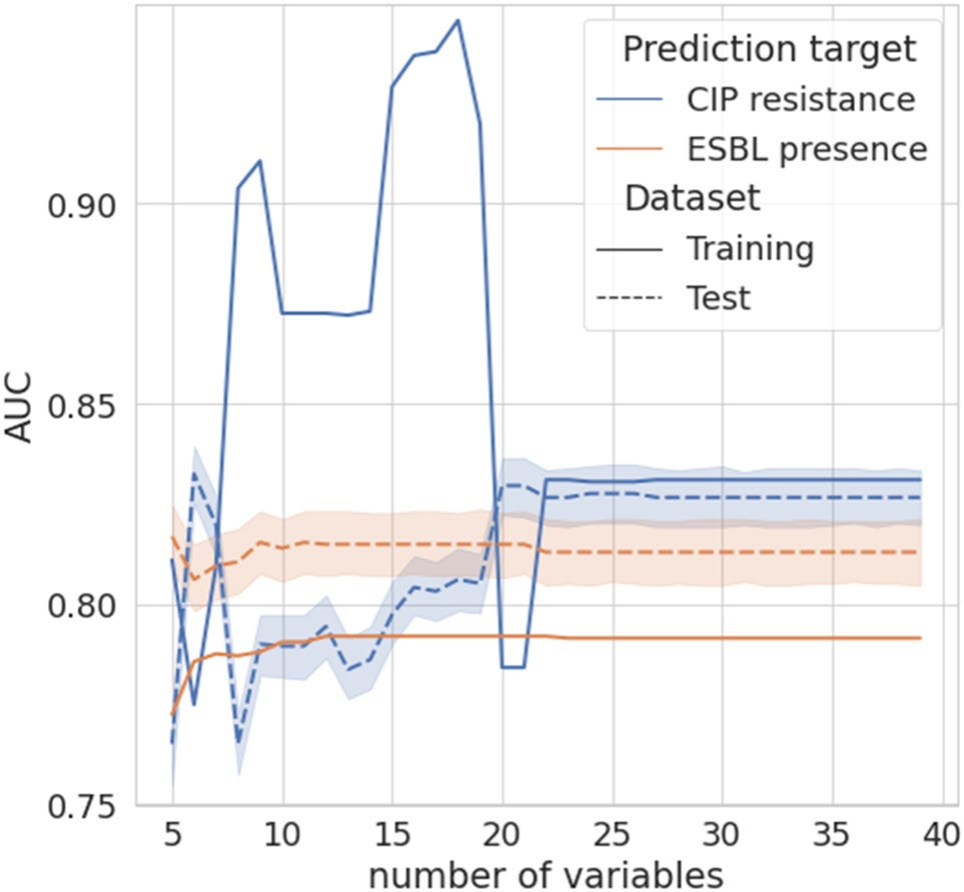
Performance according to the number of predictors. Average AUCs with 95% confidence intervals (colored region) for predicting CIP resistance (blue) and ESBL positivity (orange) each for training (solid lines) and testing (dotted lines) sets were shown.

Secondly, thirty-nine variables used in the original model were reduced in numbers through feature selection. Figure 6 showed changes in AUC according to the number of variables used. Variables were selected in order of SHAP feature values from highest to lowest as shown in Fig. 5. Because the predictors with high importance are different in predicting of CIP resistance and ESBP presence, we conducted feature selection separately, and measured each performance change according to the decrease of the number of variables. As for the prediction of CIP resistance, AUC was not significantly decreased until the number of variables was reduced to twenty compare to that of the original model with 39 variables, and there was no significant change in AUC difference between the training and test sets. Whereas, the customized model for predicting ESBL positivity showed better generalizability during variables reduction, and the performance was improved by about 1% when only five variables were used compared to the AUC of the original model. Consequently, we could narrow the number of significant predictors down to twenty and five, respectively, for predicting resistance to ciprofloxacin and the presence of ESBL while maintaining similar predictive performance. Selected variables with data distribution were summarized in Table 2.

### Performance of the models compared to empirical treatment

**Figure 7:**
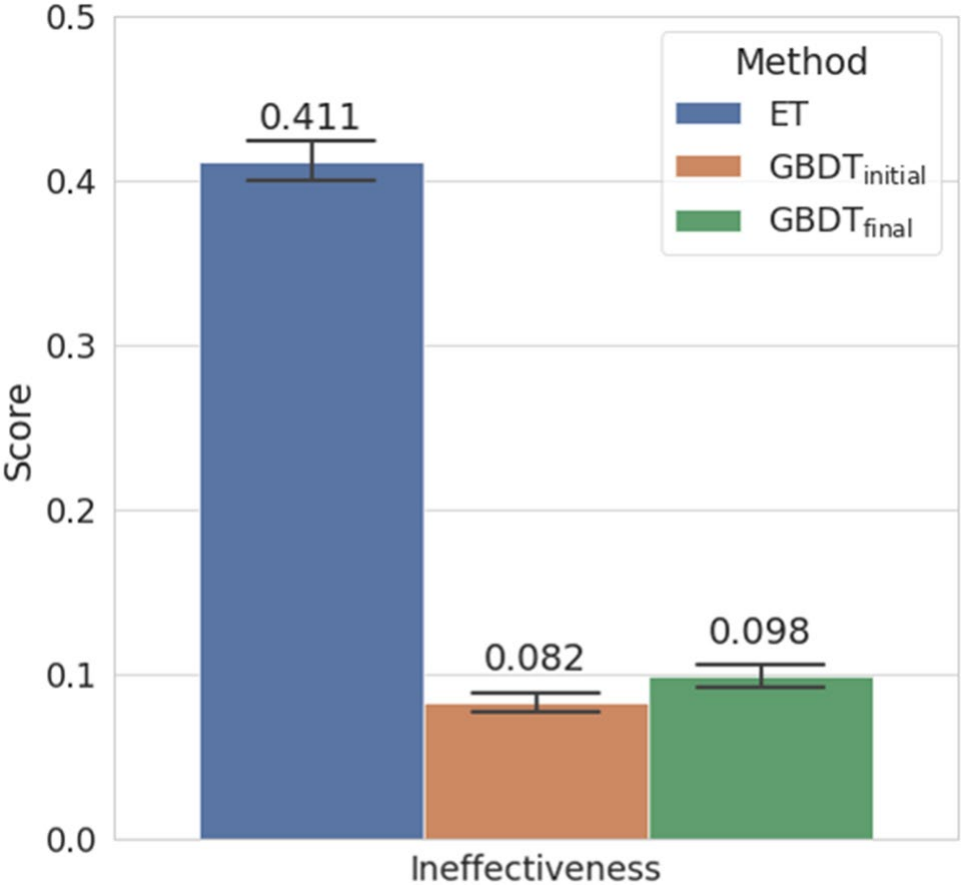
The performance of the prediction models compared with the empirical treatment (ET) regarding ineffectiveness. The initial GBDT model used all predictors and its decision threshold was set to 0.300 here. The final GBDT model used the reduced number of predictors and its decision threshold was set to 0.335. 95% confidence intervals (error bars) were obtained by bootstrap analysis.

We compared the performance of the final customized model with that of ET focusing on IE. The initial GBDT model used all predictors and its decision threshold was initially set to 0.500 then 0.300 during the first step of customization. Whereas, the final GBDT model used the reduced number of predictors and its decision threshold was finally set to 0.335. Compared to the initial GBDT model, the final GBDT model showed increased AUC, precision and specificity (Table 3). Figure 7 showed that final GBDT model lowered the probability of using ineffective antibiotics by 20% compared to the initial prediction model and ET, which was statistically significant (*P* < 0.05). This suggests that receiving GBDT support for empirical treatment in the ED could reduce the likelihood of ciprofloxacin usage in patients with ciprofloxacin-resistant uropathogens and using cefotaxime/cefepime in ESBL-positive ones.

**Table 3:** The classification performance of the prediction models compared with the empirical treatment (ET).

Prediction target	Method	AUC	Precision	Sensitivity	Specificity
CIP resistance	ET	0.502 (0.013)	0.480 (0.009)	0.900 (0.008)	0.103 (0.007)
	${\mathrm{GBDT}}_{\mathrm{initial}}$	0.827 (0.007)	0.550 (0.009)	**0.968** (0.005)	0.273 (0.011)
	${\mathrm{GBDT}}_{\mathrm{final}}$	**0.829** (0.007)	**0.555** (0.010)	0.934 (0.006)	**0.312** (0.011)
ESBL positivity	ET	0.510 (0.011)	0.530 (0.011)	0.594 (0.013)	**0.566** (0.012)
	${\mathrm{GBDT}}_{\mathrm{initial}}$	0.813 (0.008)	0.461 (0.009)	**0.961** (0.006)	0.074 (0.006)
	${\mathrm{GBDT}}_{\mathrm{final}}$	**0.817** (0.008)	**0.587** (0.011)	0.945 (0.007)	0.452 (0.012)

The initial GBDT model used all predictors and its decision threshold was set to 0.300. The final GBDT model used the reduced number of predictors and its decision threshold was set to 0.335. 95% confidence intervals (values in parentheses) were obtained by bootstrap analysis. Values in bold are the highest value across the different methods for the same target.

## Discussion

ML is a growing field in medicine, including in infectious disease (ID). By July 2019, there have been 60 ML-clinical decision support system (ML-CDSS) that have been developed to assist ID clinicians^19^. Among them, four (7%) addressed the prediction of antibiotic resistance, and two of them analysed demographic data and medical history for personalized prediction of antibiotics susceptibility or resistance. Herein we also showed that ML in combination with clinical data could improve the effectiveness of empirical antibiotic therapy in the ED. Because of the unavailability of culture results at the time of prescription, empiric antimicrobial therapy could benefit the most from ML-CDSS as our study showed. Specifically, the support of our GBDT model could help emergency physicians prescribe ciprofloxacin in UTI patients with ciprofloxacin-resistant bacteria as well reduce the likelihood of using cefotaxime/cefepime in ESBL-positive uropathogens.

There are a few strengths which set our study apart from previous studies. First, our study was based on a clear understanding of medical perspectives through the decision-making. For instance, ID clinicians begin to assess a febrile patient with the primary objective of finding the focus of fever. The possible pathogens and antibiotics with the proper coverage are only considered after the clinical diagnosis is made. As such, focusing only on individual antibiotics or pathogens using ML algorithms without a provisional diagnosis would be of no use to support clinical decisions. Therefore, we clearly defined the clinical scenario as UTI in the ED in order for the model to be successfully integrated into clinical practice. We believe this practicality which enables the real users (emergency physicians) to apply the predictive model immediately to the clinical field would be the main contribution of our research.

Under the premise of clinical diagnosis, ML-CDSS is constrained by comprehensiveness and quality of the clinical data used for their development. In this context, we analysed all available data from different sources, such as structural clinical data, vital signs, and laboratory data and our variables with high feature importance were found to be consistent previously identified risk factors^20^. We also included relevant unstructured data such as free clinical texts, nursing notes, and medical imaging to ensure the integration of detailed medical history as previous studies that analysed the performance of ML-CDSS found that the sensitivity and specificity of ML-CDSS were systematically better when they used a larger set of variables, especially when unstructured data are added^21–23^. However, data should be easily entered into the CDSS in the future system by automatic extraction from the EMR, and progress in natural language processing may help^24^. Moreover, we distinguished cases of bacterial colonisation or contamination in urine cultures from true pathogens and excluded them from the study to improve the quality of the data. These efforts to maximize the comprehensiveness and quality of our data contributed to the development of a satisfactory model in our study.

An additional important strength of our work is that algorithm training and evaluation were performed on different data sets. Then, we customized the decision threshold on the purpose of minimizing the IE. Finally, given that the labour-intensive and time-consuming process of data identification, we optimized the number of significant predictors and showed the possibility of designing an efficient model only with selected high-importance predictors while maintaining the model performance. For reference, the average elapsed times for the (1) training process including random search-based hyperparameter selection, (2) prediction on the testing set, and (3) SHAP values measurements were 5420.2, 1.6, and 17.2 ms, respectively through parallel processing using graphic processing unit.

However, this study also has a number of limitations. Firstly, our model was built on data from a single healthcare institution within a confined geographic region; thus, further validation at other institutions is needed. As resistance patterns can change over time, our model may also become less relevant as time passes and should thus also be periodically retrained^9,25^. Secondly, we assessed the performance of the model against real-life prescribers, which were emergency physicians. Ultimately, performance would need to be assessed prospectively for validation purpose with well-defined endpoints such as hospital days, mortality and medical costs to achieve better patient outcomes.

Lastly, each clinical setting might require different emphasis on using the model. For instance, a reduction in the inappropriate use of broad-spectrum antibiotics could be a priority in a community with a high rate of antibiotic overuse because improving antibiotic stewardship may lead to reduced costs, complications, and improved clinical outcomes^22^. However, we did not adjust the decision threshold of the GBDT model in our result regarding appropriateness of antibiotic use. The best approach should make it possible to substantially increase the proportion of patients who receive effective empiric antibiotics while minimizing the risk of developing resistance in each circumstance.

In conclusion, ML has the potential to help clinicians predict antibiotic resistance, improving the effectiveness of empirical antimicrobial treatment for UTI in the ED. When implemented in the hospital, our model could be a point-of-care decision support system to guide clinicians towards individualised antibiotic prescription.

## Data Availability

Data are available from the corresponding author upon request.
